# A Warm Welcome to the Alps—The Northward Expansion of *Trithemis annulata* (Odonata, Libellulidae) in Italy

**DOI:** 10.3390/insects15050340

**Published:** 2024-05-09

**Authors:** Gianandrea La Porta, Sönke Hardersen

**Affiliations:** 1 Department of Chemistry, Biology and Biotechnology, University of Perugia (PG), 06123 Perugia, Italy; 2 Reparto Carabinieri Biodiversità di Verona, Centro Nazionale Carabinieri Biodiversità “Bosco Fontana”, 46045 Marmirolo, Italy; s.hardersen@gmail.com

**Keywords:** climate warming, temperature, diachronic variation, range shift, MaxEnt, dragonfly

## Abstract

**Simple Summary:**

Climate warming has already changed the distribution and composition of European dragonflies, and this process is ongoing. One example is *Trithemis annulata*, which had been limited to southern Italy for over 150 years and which has expanded its range to the Po Plain in four decades. Recently, this species has established in some alpine valleys. We tracked the spread of this species by analysing all available data and modelled its preferred climatic conditions in Italy. Our results, which consider the years 1825–2023, indicate that *Trithemis annulata* has been moving northward since 1980 at a rate of approximately 12 km per year. Upon reaching the Po Plain in 2016, this rate increased to 34 km per year. Even though this species is known for its good dispersal ability, it was unable to keep up with the speed of the advancing climate. *Trithemis annulata* has expanded into new areas due to climate warming and is now established in Alpine valleys, which are potential gateways for the colonisation of central Europe.

**Abstract:**

Climate warming has already influenced the distribution, community composition, and phenology of European Odonata. *Trithemis annulata* had been confined to the southern regions of Italy for over 150 years. In only four decades, it has expanded its range and has recently been observed inhabiting several alpine valleys. A dataset of 2557 geographical distribution data points spanning the years 1825–2023 was compiled using various resources, with the aim to analyse the chrono-story of the expansion of *T. annulata*. A further aim was to investigate the climatic conditions that best explain its current and future distribution. Over a period of 43 years, the species steadily extended its northern range margin at an approximate rate of 12 km/year. Once it reached the Po Plain, the expansion accelerated to an average speed of 34 km/year. However, its northward shift lagged behind the warming climate as we estimated an average speed of 28 km/year. In the future, the area suitable for *T. annulata* is expected to significantly increase in Italy. Surprisingly, we did not observe any consistent upward shift. *Trithemis annulata* has considerably expanded its distribution due to human-induced climate warming. The northernmost populations now inhabit Alpine valleys, potential gateways to central Europe.

## 1. Introduction

Climate change is a global phenomenon, and human activities, principally through emissions of greenhouse gases, have unequivocally caused global warming, with global surface temperature reaching 1.1 °C above 1850–1900 levels in 2011–2020. Greenhouse gas emissions have continued to increase, arising from unsustainable energy use, land use and land-use change, lifestyles and patterns of consumption and production [1]. The impacts of global warming differ regionally and seasonally, and large parts of Europe are experiencing increased winter and summer temperatures as well as more days with extreme maximum temperatures [2]. There is no uncertainty that climate change and its multifarious, knock-on effects pose a grievous and escalating global threat to insect diversity [3]. The complexity of global climate change goes far beyond simply global temperature increase. It also leads to a variety of multifaceted ecological responses to environmental changes, including shifts in species distribution ranges, phenological displacements and other unpredictable cascading effects at different levels of ecosystem organisation [4]. Climate warming is already causing strong and consistent effects across insect taxa. Warm-adapted species are expanding their ranges in Central Europe [5,6], with dragonflies showing particularly strong responses to increased temperatures [6,7]. In fact, it has been shown that climate change has already altered European Odonata distribution along with the composition of their communities and phenologies [5,8,9].

Many authors have suggested that Odonata are ideally suited to study the effects of climate change [4,9,10], and the effects of recent climate warming have been investigated in a large number of publications [5,7,9,11,12,13]. Because species range shifts primarily depend on thermal niche, geographical ranges are expected to shift as a response to warming [10]. However, the various species differ considerably in thermal tolerances [14]. As temperature increases, higher latitudes and elevations are expected to warm, hence receiving more warm-tolerant species [15]. It is in keeping with this hypothesis that in Europe, many Odonata species have already expanded their distribution northward [5,7,9,13]. In South Korea, Shin et al. [11] documented the northward expansion of *Ischnura senegalensis* between 1980 and 2020. Additionally, in mountain regions, a shift towards higher altitudes is expected in response to climate warming [16,17]. The consequences of future climate warming have been addressed for the distribution and diversity of dragonflies. For example, Cadena et al. [18] found that in West and Central Asia, faunal composition is predicted to show strong shifts, with the increase of Oriental and Afrotropical species. Similarly, Pélissié et al. [19] reported for Northern Europe that climate change will likely result in northward range shifts of many species. Furthermore, Li and Park [10] reported that European Odonata will likely undergo significant changes in the future because of climate change. According to Jaeschke et al. [20], many protected species will lose more than 50% of their climatically suitable areas and additionally Cancellario et al. [17] forecasted widespread rearrangements in Odonata community composition as a result of climate change in Europe.

Generally, there is a large variation in the rates at which the range boundaries of individual species are moving [7,21,22]. One reason is that habitat availability can constrain expansion [20,22], and species with greater dispersal potential (e.g., larger body size or more migratory behaviour) are expected to show larger range shifts [21,23]. Among Odonata, Anisoptera are more prone to overall range increase when compared to the poorly dispersing Zygoptera [24] and species confined to lentic habitats are supposed to have a stronger propensity for dispersal than lotic species because lentic habitats are less stable in space and time [25,26,27] and dispersal propensity should thus be higher for lentic species [25,26]. Additionally, the rapid climate warming results in dragonflies accumulating a substantial “climatic debt”, which means that they cannot catch up with the poleward movement of isotherms [5,10].

The distribution of species we observe today in Europe reflects a long history of repeated glacial advances and retreats and climate warming after the last ice age, which resulted in rapid post-glacial expansion of many Odonata species (e.g., [28,29]). In a world where environmental changes occur at an increasing speed, the study of species dispersal has gained growing relevance. Thus, the analysis of the recent rapid range expansion of a dragonfly, for which a considerable amount of distributional data is available, will likely shed some light on the speed, mechanisms and direction of such a distributional shift. These data are important but, unfortunately, detailed accounts of the expansion of single species are rare in the literature (e.g., [11,13,30]). However, detecting changes using fragmented samples of data and identifying potential causes for those changes is challenging, and there are considerable uncertainties regarding the speed of distributional shifts [15]. Moving forward in the capacity to assess the where, when, and why of climate change effects on biodiversity is crucial for future strategies [15].

The present study focuses on *Trithemis annulata* (Palisot de Beauvais, 1805), a species belonging to the family Libellulidae, one of Africa’s most widespread and numerous dragonfly. It dominates open freshwater in Africa and adjacent Eurasia and, in recent decades, has spectacularly expanded its range in southwestern Europe [31,32,33,34,35]. The genus *Trithemis* dominates many freshwater habitats in Africa because of the flexible responses of these species to the climatic fluctuations since the late Miocene. Their success seems to be related to the origin of extensive savannah, which favoured opportunistic species and their dispersal ability [31]. In Italy, *T. annulata* had been confined to southern regions in Italy for more than 150 years, from the earliest published record in 1825 up to 1985 when Carchini et al. [36] reported the presence of this species for the regions Basilicata, Calabria, Campania, Lazio e Puglia, as well as for Sicily and Sardinia. Thereafter, *T. annulata* started to expand its distribution northward, and this is well documented in the literature. The species was first observed in Tuscany in 1990 [37] and in Emilia-Romagna in 2007 [38]. The first record of *T. annulata* for the Umbria region is from 2011 [39], and in 2018, the species was observed for the first time in Lombardy [40,41]. It reached South Tyrol in 2023 [35] and is now present in various alpine valleys. *Trithemis annulata* is an anisopteran species which mainly lives in lentic habitats [32] and is thus expected to have a high dispersal propensity [25,26].

This paper aims to: (i) describe the chrono-story of the expansion of *T. annulata* in Italy; (ii) analyse its northward spread in the light of recent climate warming; (iii) investigate the thermal conditions that best explain the current distribution of *T. annulata* and to predict a likely future scenario for its distribution in Italy; (iv) analyse past altitudinal shifts of *T. annulata* in response to climate change.

## 2. Materials and Methods

### 2.1. Species Data

The Italian Society for the Study and Conservation of Dragonflies (Odonata.it) maintains a database documenting Odonata sightings of adult individuals from 1766 to the present day. The earliest record of *T. annulata* dates back to 1825 (Vander Linden, 1825—Lake Averno). For this species, the database contained 2811 sightings of adults at the time of extracting the records. This dataset was integrated with 1018 records from the Global Biodiversity Information Service Network Platform (https://gbif.org, accessed on 10 January 2024) and 1159 from the iNaturalist platform (https://inaturalist.org; accessed on 10 January 2024). To prevent clustered records and overlap among observations, which could have affected the accuracy of the analyses, we allocated the observations to grid square cells, each spanning an area of 1 km^2^. Subsequently, the records were divided into time periods, and only the centroid of each cell with one or more recorded sightings was used for analyses. This resulted in a total of 2557 geographical distribution data points assembled, spanning the years 1825–2023. Each centroid was also assigned an altitude derived from the NASA Global SRTM [42].

### 2.2. Northward Expansion, Range Size and Altitude

For each time period, the mean location of the two most northerly records of the species was computed. The boundary shift of the distributional range was then estimated by calculating the differences between these mean locations across time. The range sizes, quantified as the area of occupancy (AOO), were determined by counting the number of occupied 1 × 1 km grid squares.

We used the non-parametric Kruskal-Wallis rank sum test to compare the altitudes at which the species had been recorded across different time periods. Subsequently, we carried out a Wilcoxon’s pairwise test with Bonferroni correction for post hoc analysis.

### 2.3. Species Distribution Modelling

Bioclimatic trends, reflecting aspects of temperature, precipitation and their seasonality, were obtained from the WorldClim website (Version 2.1 released in January 2020) in the form of 19 bioclim variables [43,44]. The data were downloaded for Italy at the spatial resolution of 30 s (~1 km^2^), the same scale as the species resolution. To prevent excessive correlation among climate variables, which can result in model over-fitting, we detected multicollinearity by conducting a variance inflation factor (VIF) analysis, following a stepwise correlation analysis. Each climatic variable was tested in both linear and quadratic form. The species distribution was modelled using the maximum entropy software package MaxEnt (dismo package 1.3-14) [45]. This software, widely used in species distribution modelling [46,47], was trained using a list of species occurrence locations supplemented with pseudo-absence data and a set of environmental predictors as input [48]. Before executing MaxEnt, the occurrence data for *T. annulata* were randomly split into two datasets: training and testing, with proportions of 80% and 20%, respectively. The training set was employed to calibrate the models, while the testing set was used for model evaluation. Model performance was then evaluated using the area under the ROC curve (AUC) from the spatially independent testing dataset. The AUC represents the probability that a randomly selected presence site will outrank a randomly selected pseudo-absence site. An AUC of 0.5 indicates a random ranking, while an AUC of 1.0 achieves a perfect ranking. Models scoring above 0.75 are deemed potentially useful [49]. We performed a total of 1000 runs of MaxEnt, with each run randomly changing the training and testing datasets. Similarly, future distribution patterns were generated using bioclim data derived from Max Planck Institute Earth System Model (MPI-ESM1.2) for the time period 2021–2040 under the Standard Scenario SSP245 (emissions in RCP 4.5; [1]). Finally, we examined how distribution patterns in the future differ from those in the present, generating statistics on range changes (measured in km^2^) using the ‘biomod2’ package [50].

To estimate the rate of change of temperature from south to north, we regressed temperature values in the cells where the species had been recorded against latitude separately for the years 1980 and 2020, excluding sites located at elevations above 500 m. By calculating the difference in mean temperatures between the two years, we obtained a ΔT, which, when divided by the slope of the regression line, returns the northward shift of the isotherm line (°C year^−1^/°C km^−1^ = km year^−1^).

Statistical analysis and species distribution models were performed in the R 4.3.1 and RStudio version 2023.06.1+524 environments [51,52]. QGIS 3.34 (https://qgis.org, accessed on 14 February 2024) was used to generate the final geographical maps.

## 3. Results

**Figure 1:**
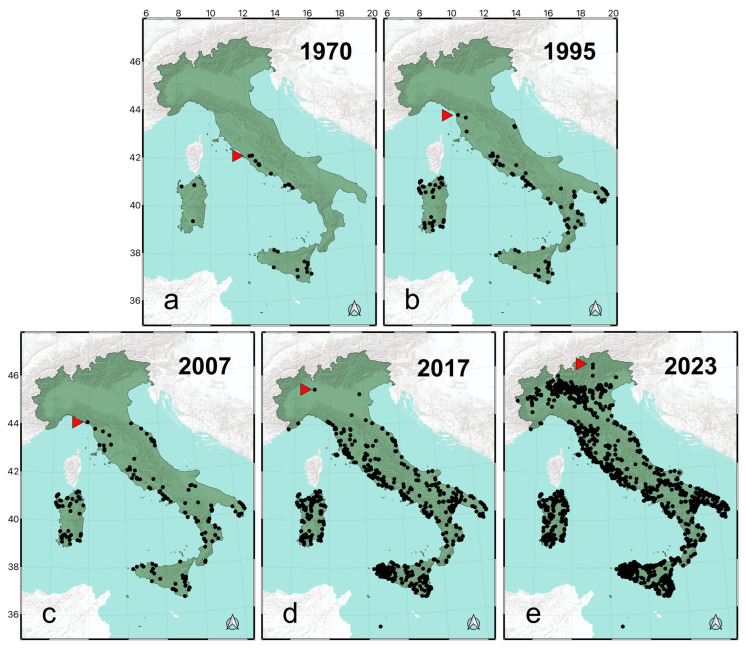
Maps show the chrono-story of the expansion of *Trithemis annulata* in Italy. Each map contains all records up to the year indicated. Records of *T. annulata* are presented for the following time spans: 1825–1970 (**a**), 1825–1995 (**b**), 1825–2007 (**c**), 1825–2017 (**d**), 1825–2023 (**e**). Red triangles highlight the northernmost records.

**Figure 2:**
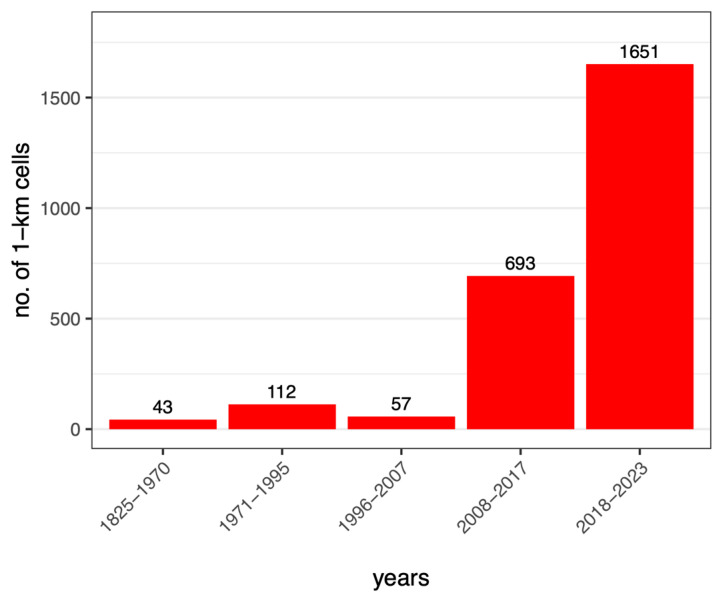
Number of 1 km^2^ grid square cells with records of *Trithemis annulata* over time.

Over time, records of *T. annulata* showed a clear progression from south to north (Figure 1). Until 1970, the species had only been documented in 43 cells in the southwestern Italian territory (Figure 2). During that period, it was restricted to the Mediterranean coasts of the Tyrrhenian Sea and did not extend above latitude 42° N (Figure 1a). In the following decades, populations were reported also from Puglia, the mainland areas of Southern Italy, and *T. annulata* was first recorded in Tuscany (below latitude 44° N), as well as along the Adriatic coast of the region Marche (Figure 1b). Subsequently, the number of records increased progressively, and the species’ range expanded for the first time beyond latitude 44° N (1996–2007) (Figure 1c) and latitude 45° N (2008–2017). *Trithemis annulata* reached the inland area of Milan by 2017 (Figure 1d), and in this time period, an additional 693 cells were added to the territory known to be occupied by the species. In the period 2018–2023 (Figure 1e), the number of records grew exponentially, representing an increase of 82.4% in data compared to the records collected over nearly two centuries (Figure 2).

**Figure 3:**
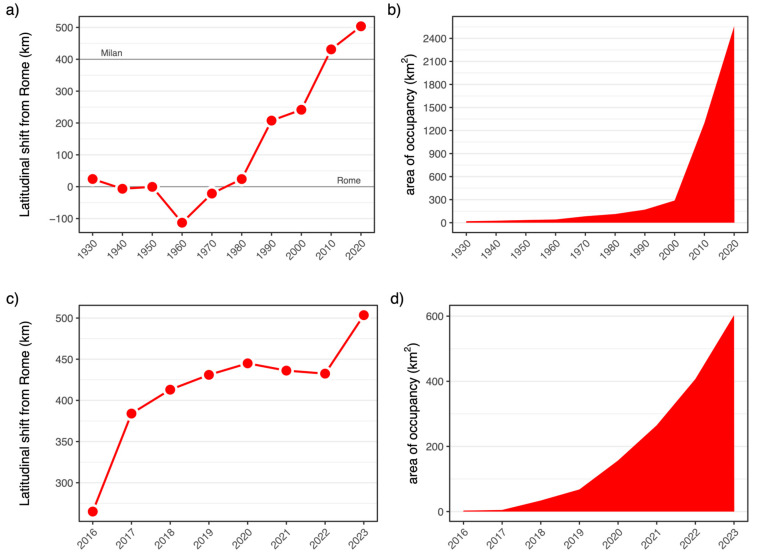
Latitudinal shift and variation of occupancy of *Trithemis annulata* in Italy since 1930 (**a**,**b**), and in Northern Italy since 2016 (**c**,**d**).

The analysis of the latitudinal shift over 10-year periods revealed that until the 1980s, the species did not exceed the latitude of Rome (at 42° N) (Figure 3a). Subsequently, a phase of northward progression began, characterised by a shift of the northern range margin of approximately 12 km/year. Additionally, the species experienced an important increase in range size, leading to an important expansion of its area of occupancy (>700%), accelerating from the turn of the century (Figure 3b).

Starting in 2016, a year-by-year analysis of the shift of the range margin in northern Italy revealed two important advances (Figure 3c). The first significant northward leap is represented by a record in the Milan area in 2017. The second important advance in the northern direction is the expansion along the Adige Valley in the Alps in 2023. During the intervening years (2018–2022), a slower northward progression was evident, marked, however, by a significant increase in range size (Figure 3d).

**Figure 4:**
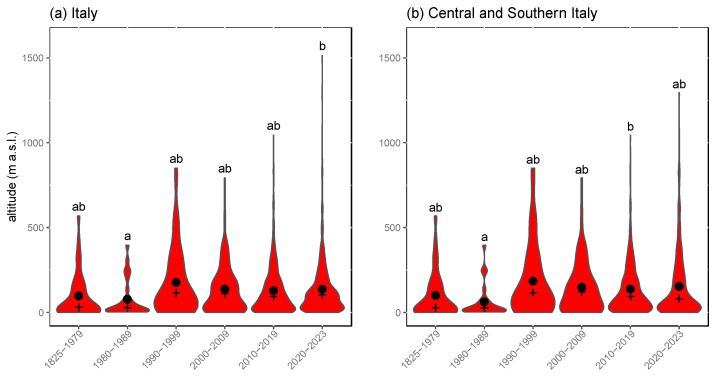
Violin plots illustrating the altitudinal distribution of *Trithemis annulata* over time in Italy (**a**) and in Southern-Central Italy (**b**). Black dots represent mean values, and plus signs represent median values. Distinct letters indicate statistically significant differences.

Surprisingly, no consistent upward shift in altitude was detected for *T. annulata* from 1825 to 2023 (Figure 4). The median altitude of records ranged from 17 to 110 m a.s.l, with slight differences between the 1980s and 2020–2023 (*p* < 0.05). However, the medians for these time periods were not significantly different when only the data for southern Italy were analysed (Figure 4b). Even though the species was mainly recorded at low altitudes (<500 m a.s.l.), the number of records at higher elevations has consistently increased, and after the year 2010 *T. annulata* has been recorded also above 1000 m a.s.l in the Alps, as well as in Central and Southern Italy (Figure 4).

**Figure 5:**
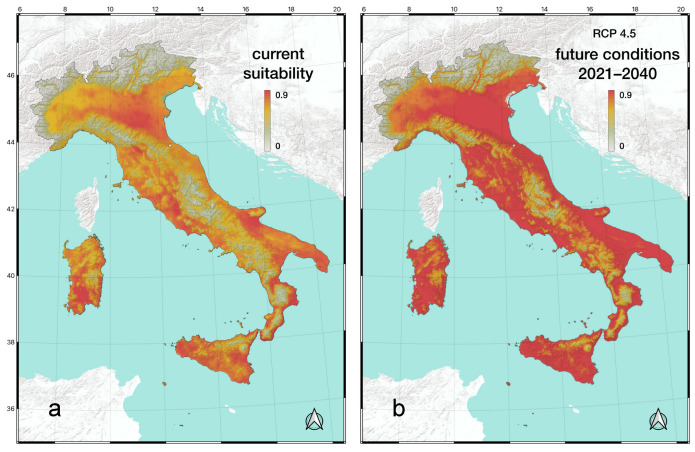
Climate suitability maps for *Trithemis annulata* under current conditions (**a**) and future scenario (RCP 4.5) (**b**).

The MaxEnt model was used to identify the most important factors explaining the distribution of *T. annulata* in Italy and to predict the areas potentially suitable under current and future climatic conditions (Figure 5). The overall performance of models was good, with an average AUC of 75.5% (CI 75.2–75.9%). After setting up and running the MaxEnt model, we used the jackknife method to evaluate the importance of the different environmental factors. The analysis showed that bio5 (Max Temperature of Warmest Month) was the most important factor (96.8%), while the other two variables, bio3 (Isothermality) and bio14 (Precipitation of Driest Month), contributed only 2.7% and 0.5%, respectively. Based on the MaxEnt modelling results, we plotted the current potential distribution map of *T. annulata* in Italy (Figure 5a). The light grey areas were not suitable for the species (0.00–0.25); the light green areas indicated a low-suitability (0.25–0.45); the yellow areas corresponded to a mid-suitability (0.45–0.65); and the orange-red areas represented highly-suitability areas (0.65–0.90).

Under climate scenario SSP 2–4.5, and using the selected bioclimatic factors, Italy is expected to experience an expansion of territory highly suitable for *T. annulata* from the currently 100,718 km^2^ to 302,801 km^2^ for the time period 2021–2040, representing an increase of approximately 200%. Obviously, this climate model does not consider the distribution of potential habitats for *T. annulata* (Figure 5b).

In Italy, the maximum temperature during the warmest month showed a statistically significant linear pattern from south to north, with an average decrease of 0.21 ± 0.03 °C for every 100 km of latitude (r = −0.75; *p* < 0.0001). When comparing temperatures between 1980 and 2020, a significant increase was detected (t = −14.205, *p* < 0.0001), with a mean value of 2.38 ± 0.33 °C. This difference in temperature corresponded to a northward shift of the isotherm line of 1118 (±130 km) over 40 years, which translates into a rate of 28 km/year (Figure S1).

## 4. Discussion

This study investigated the chrono-story of the expansion of *T. annulata* in Italy, which began in the southwest, where the species was confined from 1825 to 1970 (Figure 1). The species first expanded its range to the southern tip of Puglia, but until 1980 did not show any important northward expansion (Figure 3). Its distribution shifted to higher latitudes for the first time in the early 90’s, when the species was first recorded in Tuscany [37], and this expansion continued up to 2023, when *T. annulata* reached South Tyrol [35]. The species also reproduced in the newly colonised areas, as evidenced by the finding of exuviae [40,41].

Over this 43-year period, the species extended its northern range margin at an average rate of approximately 12 km/year (Figure 3). This progression occurred mainly along the Tyrrhenian and Adriatic coast, but once the species had reached the Po Plain, the expansion did no longer follow the coastline. Here, the species advanced with an average speed of 34 km/year. The distribution of *T. annulata* was well described using the MaxEnt model, and the factor bio5 was by far the most important one. Its isotherm advanced at a rate of 28 km/year. As expected, the progression of this isotherm is particularly rapid, given that summer extreme temperatures rise much faster compared to winter or annual mean temperatures [53]. Thus, our data showed that the distribution of *T. annulata* can be well explained with a climate model and that the northward expansion of this species is in line with a northward shift of the most important climatic variable.

Our results are in line with Ott’s [30] findings on the colonisation of Germany by *Crocothemis erythraea* in about four decades. Also, in this case, the fast expansion reflected the change in the climatic conditions, and that northward shift showed a similar magnitude to *T. annulata* described here. The speed of the advancing northern boundaries has been estimated only for a few Odonata species. For example, Greve et al. [26] investigated recent range shifts of European dragonflies and found that lentic dragonflies of the southern group shifted their range boundaries most, advancing at a rate of approximately 5.8 km/year. Platts et al. [22] found for Britain that *Anax imperator* has expanded polewards at 10 km/year in response to climate warming, and Shin et al. [11] reported for South Korea that the northern range boundary of *Ischnura senegalensis* shifted at a rate of about 11.2 km/year. Thus, *T. annulata* is advancing rather fast in Italy, particularly considering the 34 km/year observed over 7 years in northern Italy. However, for its congener *Trithemis kirbyi* Gil-Tapetado et al. [13] reported that this species has a high dispersal potential and can disperse an average of 430 km per year and this ability to cross large distances quickly has been confirmed by sightings of this species on isolated islands in the Mediterranean basin [54]. When considering Odonata in general, the advance of the northern limit is slower. For example, Hickling [55] found for British Odonata that southern species shifted northwards at the range margin by approximately 3.52 km/year, a slightly lower expansion rate has been reported by Pélissié et al. [19] for Northern European odonates, with a projected average latitudinal shift of 1.83 to 3.25 km/year.

The remarkable increase in occupancy was caused by two factors: firstly, many more odonatological records being collected over the last 20 years, and secondly by the northward expansion of *T. annulata*. This invasion of northern Italy resulted in many aquatic habitats being colonised by *T. annulata*. In a large number of wetlands in northern Italy, this species is now one of the most common dragonflies (pers. obs). The consequences of such an addition for local communities have not been investigated, but a decline in some species which occupy a similar niche seems inevitable [30,56]. Bonet Betoret [57] provides some examples of *Crocothemis erythraea* becoming much scarcer after the arrival of *T. annulata*. Thus, it seems likely that the addition of *T. annulata* to freshwater habitats in northern Italy will be to the detriment of other species and that, generally, the addition of advancing southern species to northern freshwater ecosystems will considerably change local communities. Such changes in the distribution of dragonflies and damselflies are expected all over the world [17,18,58,59].

If we consider the expansion from 1980 to 2023 of *T. annulata*, the rate of the northward shift is still lagging behind the advancing temperature. We estimated an average of 28 km/year for the advancing isotherm of maximum temperature in the warmest month, and *T. annulata* is thus accumulating a “climatic debt”, as it moved at an average rate of “only” 12 km/year for 40 years. This is in agreement with the finding that generally, in Europe, dragonflies are accumulating a substantial climatic debt [5], and other taxonomic groups, such as birds and butterflies, do not keep up with temperature increases [60].

It is clear that this climatic niche modelling approach assumes that range changes are determined solely by the availability of climatically suitable habitat, without additional limitations imposed by dispersal or life history [23]. In the case of *T. annulata*, the shift of its distributional range is expected to occur relatively quickly because it belongs to the group of southern lentic Anisoptera species and is also a Mediterranean-afrotropic thermophilic dragonfly. These species are characterised by a very high dispersal ability [25,26,61,62]. Additionally, the species belongs to the genus *Thithemis*, a genus that has shown flexible responses to climatic fluctuations since the late Miocene. These species are considered opportunistic and have good dispersal ability [31]. Moreover, it seems likely that habitat availability was not a major constraint to the expansion of *T. annulata*, as this species breeds in a wide range of sun-exposed, slow flowing and standing waters, such as quarry lakes, small basins, natural lakes and sluggish streams and rivers [32,35,40,41] and these habitats are very common along the coast and in the Po Plain [63]. Despite this, we found that *T. annulata* was not able to keep up with the poleward movement of the isotherm associated with the bioclimatic variable that best explained its distribution. The speed at which species can track a moving climatic niche might be influenced by many aspects. For example, if individuals are more likely to emigrate at high densities, range expansion is slowed as newly colonised sites emit few emigrants until their population sizes have built up [64], and this would limit the speed of the expansion of *T. annulata*. Importantly, most species are probably less well equipped to advance rapidly northwards when compared to *T. annulata* as traits such as dispersal ability, body size, wingspan, etc. affect species range shifts as a response to climate change [10] and it is thus not surprising that dragonflies in general in Europe are accumulating a substantial climatic debt [5]. The above-mentioned intrinsic factors interact with other drivers, such as fragmented habitats, which may reduce the probability of species dispersal to new areas [10] also, the distance between suitable habitats is important for the ability of species to track climate change. If suitable dispersal corridors are absent, species responses to climate change cannot be realised [20,22,24]. Therefore, habitat connectivity becomes increasingly important for survival [4] as range shifts in response to climate change are often constrained by insufficient habitat connectivity in fragmented landscapes [22].

Employing the MaxEnt model, we found that thermal conditions explained the current distribution of *T. annulata* well and that bio5 (Max Temperature of Warmest Month) was the most important factor (96.8%). Also, the distribution of other Odonata species has successfully been modelled using MaxEnt and bio 5 is one of the most important variables for predicting species distributions of Palearctic Odonata [65]. In contrast, for *Trithemis kirbyi* the most important bioclimatic variable explaining the distribution of suitable areas was the minimum temperature of the coldest month (bio6) [13], and for *Ischnura senegalensis*, the annual mean temperature and the maximum temperature of the warmest month contributed a total of 79.9% of habitat suitability [11].

When we used MaxEnt to predict the likely future distribution of *T. annulata* in Italy, we found an expansion of the territory highly suitable for *T. annulata*, which increased from currently 100,718 km^2^ to 302,801 km^2^ for the period 2021–2040 in a warming world. It seems important to emphasise that in this future scenario also, many alpine valleys, such as the Adige Valley, will become highly suitable (Figure 5b) for this species. This is important, as the Alps are a natural barrier for the movement of many Odonata [66], and these valleys are the most likely corridors for Odonata when colonising Austria and Germany [67]. Movements through the western valleys could facilitate the connection between Italian and French populations, advancing through the Rhone Valley and Savoie. The valleys of the Central Alps are likely to provide access to the lentic ecosystems of Switzerland.

Surprisingly, in our multidimensional assessment of the range shift along all three geographic dimensions (latitude, longitude, and elevation) we did not detect a consistent upward shift of *T. annulata* from 1825 to 2023, even when considering only central and southern Italy (Figure 4b) and we are unaware of any reports on Odonata species that have clearly shown uphill movements in response to climate change. However, modelling approaches found that the preferred mean elevation under future climate scenarios will be higher for European Odonata and for dragonflies in South Africa [17,59,68]. Our climatic niche modelling approach assumed that range changes were determined solely by the availability of favourable climatic conditions [23], but it seems likely that suitable habitats for *T. annulata* become more sparse with increasing altitude. Even though adults were often observed above 1000 m a.s.l. after the year 2010 (Figure 4), the large majority of occupied cells was located below 250 m a.s.l. and this has not changed over the time span investigated. In contrast, uphill shifts in butterflies, Coleoptera and other terrestrial insect groups have been reported as a response to climate change [69,70,71]. In these cases, mountains presumably provide continuous habitats for terrestrial species, and consequently, these insects can follow their preferred temperatures upwards as the climate is warming. In contrast, the aquatic habitats available often change considerably when moving up a mountain, and therefore, uphill shifts in Odonata populations are much more constrained.

An important criticism about studies focusing on climate-induced range expansions is that detected expansions are simply the outcome of a higher number of records in the following observation period [26]. It is a common problem to combine relatively little data from before the year 2000 with a large quantity of records gathered by citizen scientists (e.g., [11,72]). However, we are confident that the reported northward expansion is not an artefact for a number of reasons. Firstly, *T. annulata* is a species with a striking appearance, and even beginners recognise it immediately when present in water, an argument also applied by Ott [30] for *Crocothemis erythraea*. Secondly, we do have historical, reliable data for many of the sites in northern Italy where the species is now common (e.g., [73,74]), and therefore, the presence of *T. annulata* would not have remained unnoticed here. Thirdly, the data presented here coincide with the chrono-sequence of many first records of *T. annulata* published for Italian regions [35,37,40,41]. Fourthly, the northward shift reported here is in line with the expansion of *T. annulata* in other European countries, such as Spain, France, Slovenia and Croatia [33,34,57,75,76].

*Trithemis annulata* has expanded its distribution due to human-induced climate warming. It is, therefore, difficult to decide if this species should be considered native or alien in northern Italy. Essl et al. [77] suggested applying the new term neonative to species that have expanded geographically beyond their native range and have established populations whose presence is due to human-induced changes in the biophysical environment but not as a result of direct movement by human agency, and this definition certainly applies to *T. annulata*.

## 5. Conclusions

Our comprehensive analysis of the expansion of *Trithemis annulata* in Italy retraces the chrono-story of its northward progression. From its confined origins in the southwest of Italy, *T. annulata* steadily expanded its range along two of three geographic dimensions, eventually reaching South Tyrol by 2023. Concomitantly, the number of cells with records of this species increased enormously. The northern range margin of *T. annulata* advanced at a swift pace, averaging 12 km/year over 43 years, with an even more accelerated rate of 34 km/year observed over 7 years in northern Italy. Comparisons with similar studies on dragonflies across Europe underscore the remarkable pace of the expansion of *T. annulata*, and this reflects its good dispersal capability and adaptability under changing climatic conditions. However, despite its abilities and the abundance of suitable habitats, *T. annulata* struggled to keep pace with the northward shift of human-induced climatic variables. As *T. annulata* continues its northward expansion, it will colonise new freshwater habitats and compete with native taxa as well as with introduced species. In a rapidly changing world, it is important to understand the dynamics of species dispersal, and the fast expansion of *T. annulata* represents an instructive example.

## Data Availability

The script, the model outputs, and the public occurrence data are available at the request of the authors.

## Supplementary Materials

The following supporting information can be downloaded at: https://www.mdpi.com/article/10.3390/insects15050340/s1, Figure S1: South–north gradient of the bioclimatic variable bio5. Data points represent temperature records at sites where *Trithemis annulata* had been recorded in 1980 or 2020.
