# Supplementary material for: A Warm Welcome to the Alps—The Northward Expansion of Trithemis annulata (Odonata, Libellulidae) in Italy
Source: Insects. 2024 May 9;15(5):340. doi: 10.3390/insects15050340 (PMC11121767; doi:10.3390/insects15050340)
Supplement: Supplementary file 1 [file insects-15-00340-s001.zip › insects-2960776-supplementary.pdf]

Supplementary material

A warm welcome to the Alps - the northward expansion of *Trithemis annulata* (Odonata, Libellulidae) in Italy

Gianandrea La Porta[1,*] and Sönke Hardersen[2]

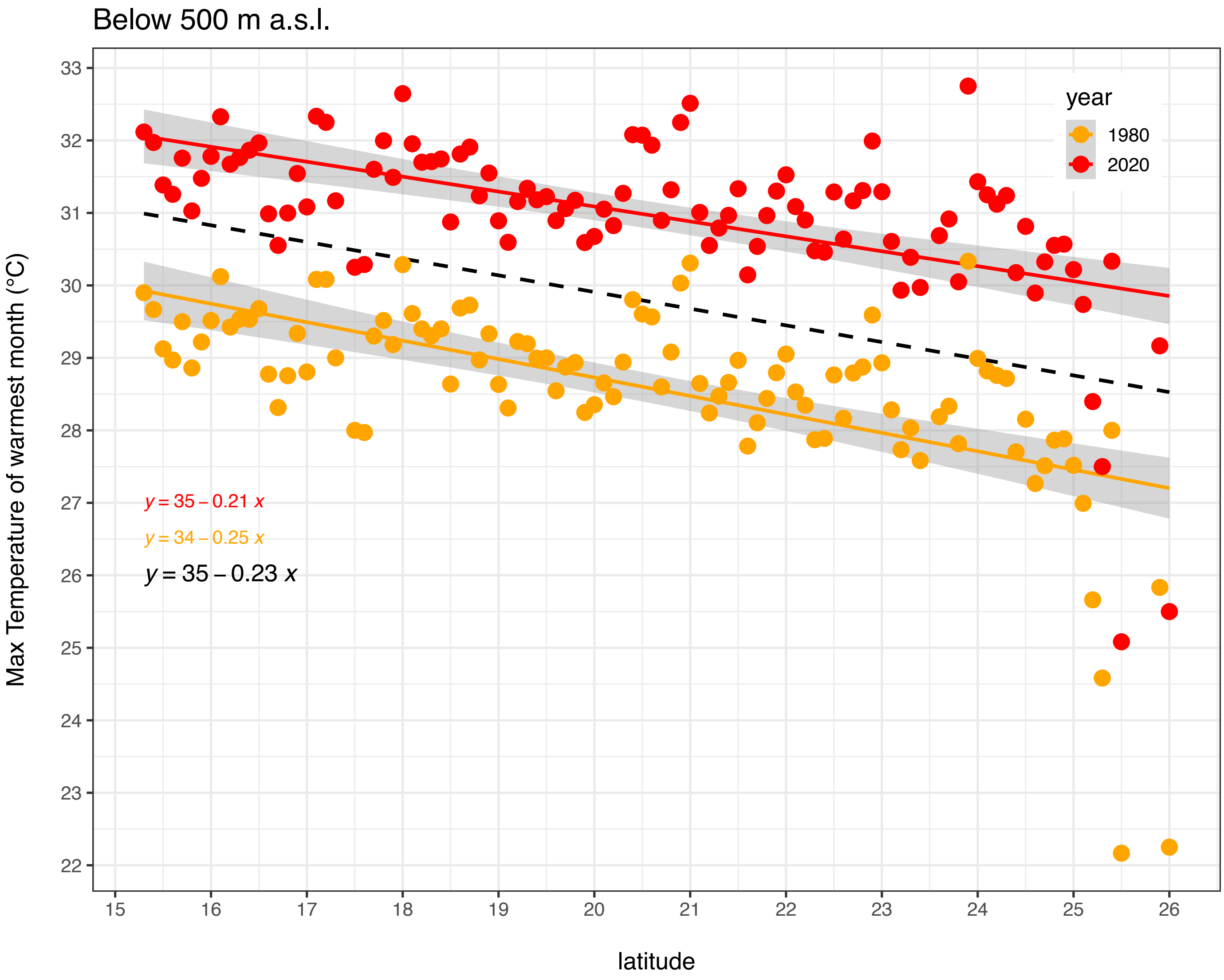

Figure S1: South–north gradient of the bioclimatic variable bio5. Data points represent temperature records at sites where *Trithemis annulata* had been recorded in 1980 or 2020.
